# Schwannomatosis Presenting With a Grade IV Glioblastoma: A Case Report and Literature Review

**DOI:** 10.7759/cureus.23381

**Published:** 2022-03-22

**Authors:** Nardine Abdelsayed, Zachary Bondranko, Peter Montesano

**Affiliations:** 1 Department of Internal Medicine, Grand Strand Medical Center, Myrtle Beach, USA

**Keywords:** lztr1, smarcb1, neurofibromatosis, grade iv glioblastoma, schwannomatosis

## Abstract

Schwannomatosis is a rare subset of neurofibromatosis. It is a disease process with a predisposition to schwannomas in the absence of bilateral vestibular schwannomas, which differentiates it from neurofibromatosis 2 (NF2). It is occasionally associated with certain tumors such as malignant peripheral nerve sheath tumors or rhabdoid tumors. Currently, there is limited literature to suggest an association between schwannomatosis and glioblastoma (GB). We present a case of a 55-year-old female with a history significant for schwannomatosis who presented after a witnessed first-time seizure with left facial weakness and slurred speech. She was found to have a 3 cm right-sided ring-enhancing lesion that was excised and found to be a grade IV Isocitrate dehydrogenase (IDH) wildtype GB.

## Introduction

Neurofibromatosis (NF) is divided into three separate diseases: NF1 which is the most common subtype, NF2, and schwannomatosis. The hallmark of all three diseases is the presence of benign peripheral nerve tumors, although they can also be linked to various malignancies. Schwannomatosis was first recognized as a separate entity from NF2 in 1990 [1]. Patients with schwannomatosis generally present in their 20s or 30s with multiple Schwann cell tumors called schwannomas. In northwest England, it was found to occur in 1 out of 126,000 people [2]. Patients develop one or more schwannomas that may present as pain due to compression, a palpable mass, or other symptoms depending on the location. They occur most commonly in peripheral nerves, but may also present in spinal nerve roots (occurring in about 75% of patients and are usually in the lumbar spine), cranial nerves (non-vestibular), or subcutaneous nerves [3]. Spinal schwannomas usually involve the dorsal root ganglia and thus generally present with pain and paresthesia. Schwannomas are best visualized using high-resolution magnetic resonance imaging (MRI) with contrast and thin cuts (1 mm). Pathology of schwannomas shows alternating areas of compact spindle cells arranged in compact ‘fascicles’ (Antoni A areas) and fewer cellular areas (Antoni B lines) [4]. Glioblastomas have previously been associated with NF1, but are not commonly seen in schwannomatosis. We present a case of a rare occurrence of glioblastoma in a patient with schwannomatosis. 

## Case presentation

Our patient is a 55-year-old female with a past medical history of multiple spinal schwannomas requiring a spinal vagal nerve stimulator, schwannomatosis (no evidence of bilateral vestibular schwannomas on previous auditory canal MRI), Hashimoto's thyroid disease (with history of thyroidectomy) who presented with left-sided facial weakness and slurred speech that began earlier that day after a new-onset witnessed seizure. Her deficits resolved quickly except for persistent right-hand numbness. Her family history was significant for breast cancer requiring bilateral mastectomy in her mother and colon cancer in her father. Lynch syndrome was noted in the paternal side of her family. She endorsed no family history of neurofibromatosis or schwannomatosis. She denied any smoking history or heavy alcohol use.

**Figure 1:**
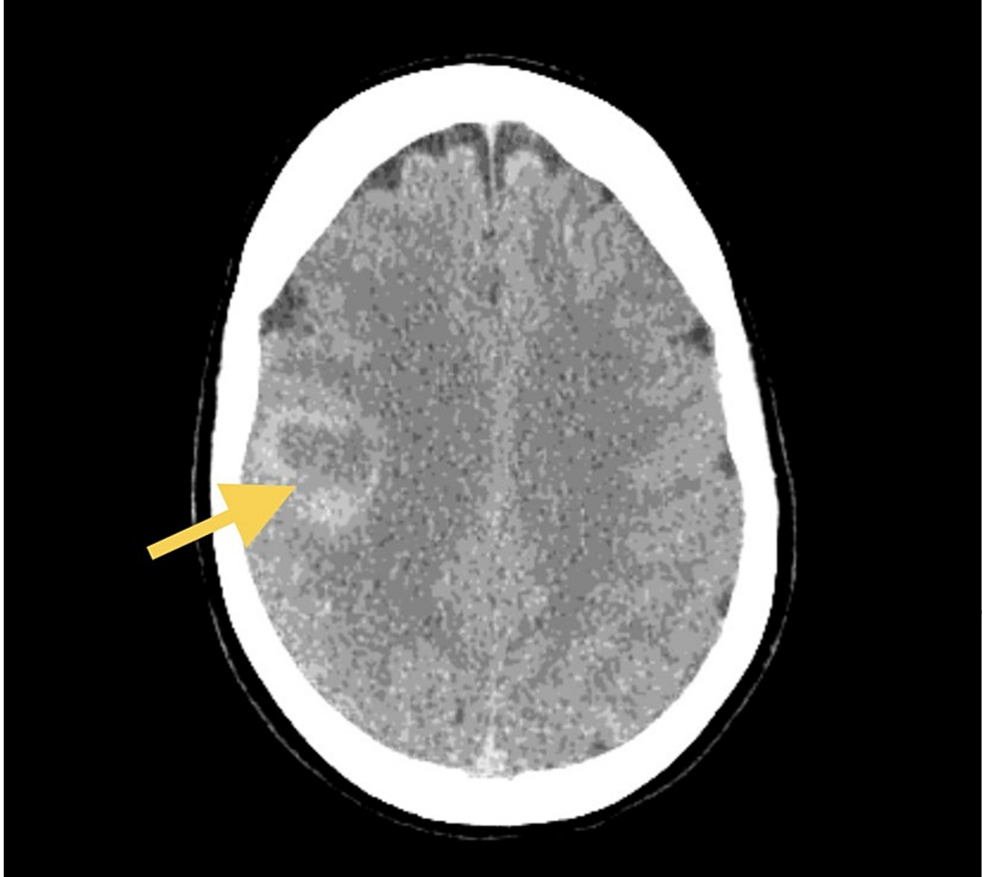
Computed tomography (CT) head with the yellow arrow showing 3.0 X 2.9 cm mass in the right cerebral hemisphere near the gray-white junction.

**Figure 2:**
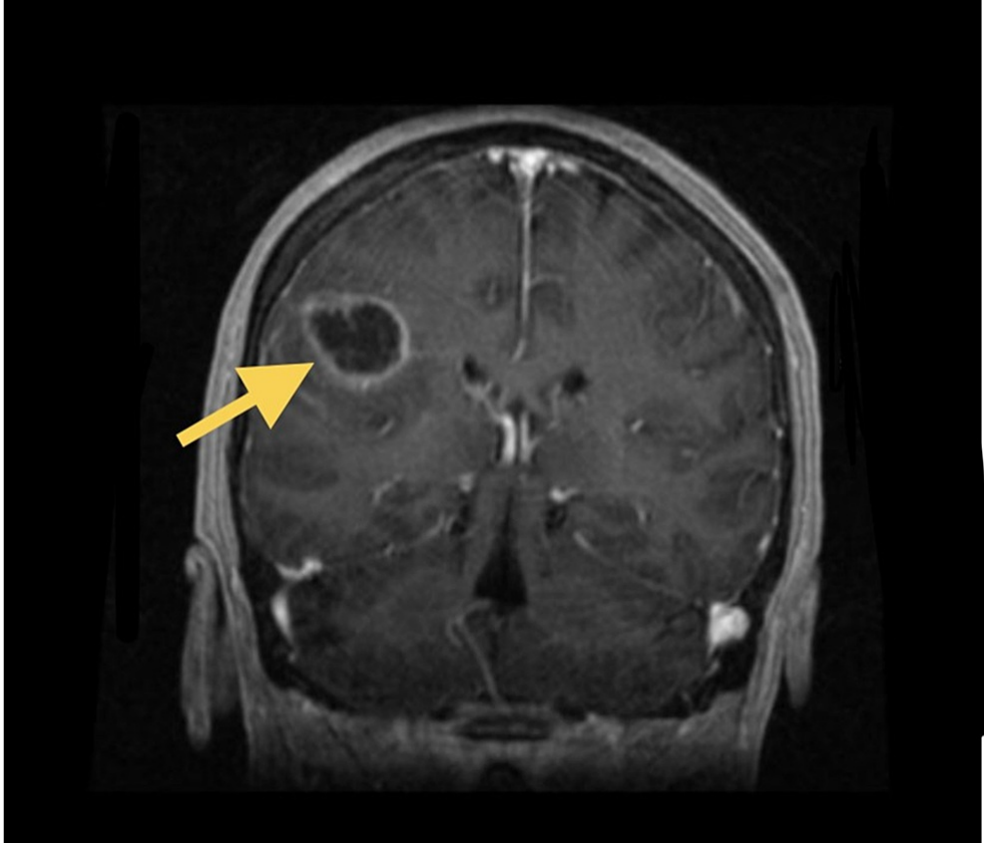
Coronal view on MRI flair with the yellow arrow showing posterior right frontal lobe mass.

**Figure 3:**
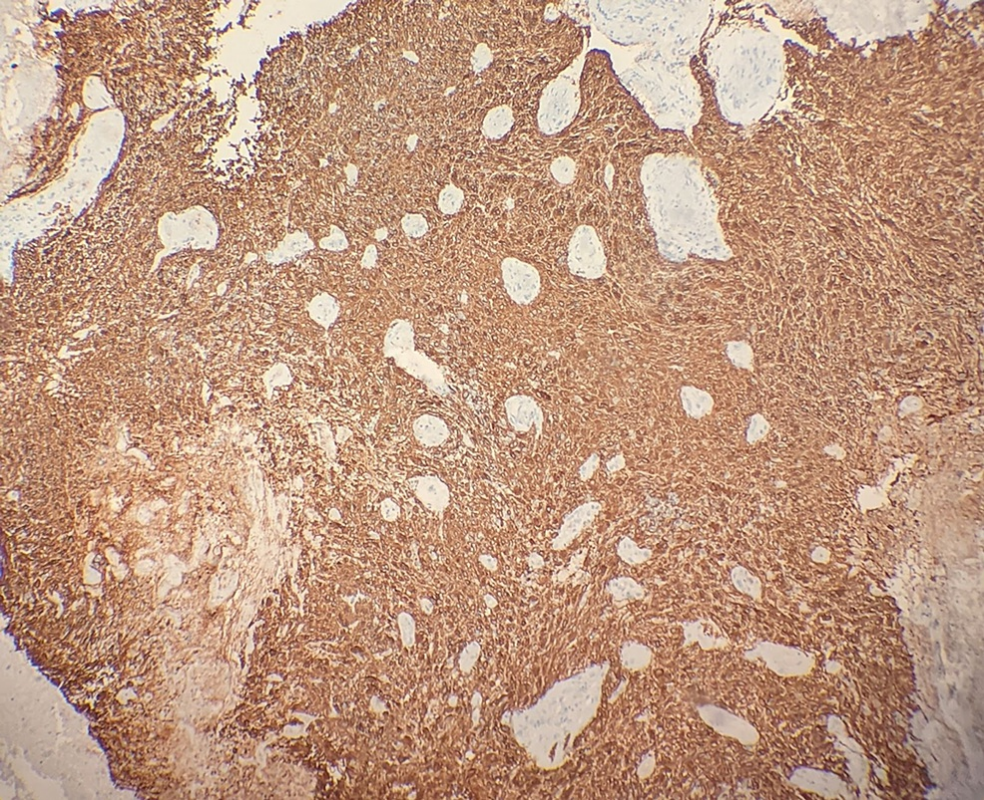
100x, Immunohistochemical analysis for GFAP is positive, consistent with a glial neoplasm.

Her original vital signs in the emergency department were normal except for an elevated heart rate at 100 beats per minute and her oxygen saturation was 95% on 2 liters nasal cannula. Her laboratory findings were notable for an elevated c-reactive protein of 1.5 (normal 0-0.99 mg/dL). Computed tomography (CT) of her head showed a 3 cm ring-enhancing lesion within the right cerebral hemisphere at the gray-white junction with mild edema and 0.3 cm right-to-left midline shift (Figure 1). Magnetic resonance imaging (MRI) showed a right frontal mass with irregular margins and central necrosis (Figures 2-3).

**Figure 4:**
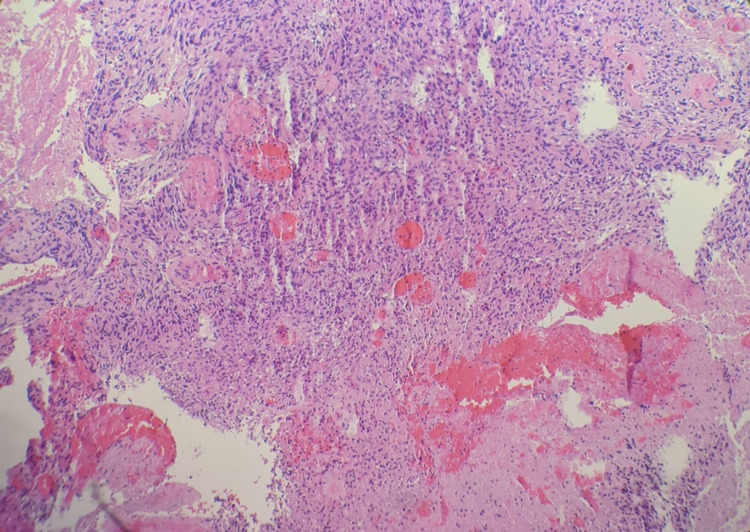
100x, Hematoxylin and eosin (H&E) stain showing hyper-cellular glial neoplasm with marked nuclear atypia, geographic necrosis, and microvascular proliferation; consistent with glioblastoma.

**Figure 5:**
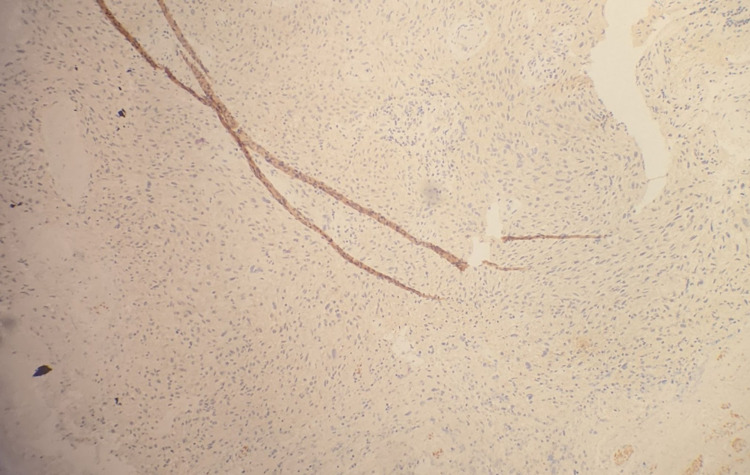
Negative immunohistochemical analysis for IDH1 R132H (no nuclear expression), consistent with a wild-type (“de novo”) glioblastoma (100x).

Neurosurgery was consulted and performed an excision of her right frontal mass and she was subsequently taken to the neurology intensive care unit for close monitoring. Her follow-up MRI showed resection of the previously mentioned mass and post-surgical changes with residual edema and 1 mm right to left shift. She was started on dexamethasone and levetiracetam for seizure prophylaxis. Surgical pathology returned positive for glial fibrillary acidic protein (GFAP) and negative for pankeratin, consistent with a glial neoplasm. Immunohistochemical analysis reveals tumor cells are negative for IDH1 R132H, consistent with a wild type ("de novo") glioblastoma (pathology slides shown in Figures 4-6).

She was monitored for 72 hours with no noted deficits and was discharged home with the continuation of dexamethasone and levetiracetam, and instructions to follow up with neurosurgery, oncology, and radiation oncology. She was started on temozolomide chemotherapy by her oncologist and began radiation therapy treatments. 

## Discussion

Diagnostic criteria for ‘definite’ (Table 1) or ‘possible’ (Table 2) schwannomatosis as defined by MacCollin et al. are discussed below [5]. The main differential diagnosis for schwannomatosis is NF2 and thus an MRI of the auditory canal is necessary to rule out bilateral vestibular schwannomas. Clinically, patients with NF2 often have visual impairment due to cataracts, optic nerve meningiomas, retinal hamartomas, and epiretinal membranes which is not common in schwannomatosis and further differentiates the disease from NF2 [6]. 

**Table 1:** Criteria for 'definite' schwannomatosis

Criteria for definite schwannomatosis
Age >30 years and all of the following:
1. Two or more non-intradermal schwannomas, at least one with histologic confirmation
2. Diagnostic criteria for NF2 not fulfilled
3. No first-degree relative with NF2
4. No known constitutional NF2 pathogenic variant
5. No evidence of vestibular schwannoma on high-quality MRI scan
OR One pathologically confirmed non-vestibular schwannoma plus a first-degree relative who meets the above criteria

**Table 2:** Criteria for 'possible' schwannomatosis *See Table 1.

Criteria for possible Schwannomatosis
Age <30 years and numbers 1-5 from the previous table*
OR Age >45 years, no symptoms of eighth cranial nerve dysfunction, and numbers 1-4 from the previous table*
OR Radiographic evidence of a schwannoma and first-degree relative meeting criteria for definite schwannomatosis

Genetic causes of schwannomatosis include two main contributing genes. First is the loss of expression of SMARCB1 (a tumor suppressor gene), which is seen in 40-50% of families with schwannomatosis [7]. The second is loss of function mutation of another tumor suppressor gene, leucine zipper-like transcription regulator 1 (LZTR1), which was seen in about 38% of patients with familial schwannomatosis and in 22% of sporadic cases [7].

Glioblastomas have been previously seen in association with NF1, but there is limited literature to suggest an association between GB with schwannomatosis [8]. Our case highlights a patient with a notable history of schwannomatosis who was found to have a grade IV IDH wildtype GB. 

Schwannomatosis has been associated with a variety of tumors. Meningiomas are seen in 5% of patients with this disorder. Malignant peripheral nerve sheath tumors are reported in 3%, and rhabdoid tumors are seen in a few cases [9]. Glioblastomas have been previously linked with NF1, but there is limited literature suggesting a link between schwannomatosis and glioblastoma. A PubMed search of ‘schwannomatosis’ and ‘glioblastoma’ only yields nine articles. Two articles did note the coexistence of these two rare conditions [10], specifically linking loss of function mutation of LZTR1 mutations. LZTR1 is a tumor suppressor gene that facilitates the polyubiquitination and degradation of RAS via the ubiquitin-proteasome pathway, leading to the inhibition of the RAS/mitogen-activated protein kinase (MAPK) signaling. Loss of function mutation disinhibits RAS/MAPK, which allows for unregulated gene expression, cell growth, division, and differentiation [11].

Light microscopy and immunohistochemistry are used for diagnosing glioblastomas. Pathology shows densely cellular, pleomorphic tumors with mitotic activity and either microvascular proliferation or necrosis, or both. Resection, adjuvant radiation, and chemotherapy all play a role in management in GB. Maximal surgical resection is generally preferred with preservation of neurological function. Glioblastoma tends to present with microscopic extensions into the surrounding brain parenchyma and is then targeted by radiation therapy, which has been shown to improve survival following surgery [12]. Testing for isocitrate dehydrogenase (IDH) type 1 or type 2 mutations is performed since patients with IDH1/2-mutant mutations tend to have a better prognosis. Our patient had the grade IV IDH-wildtype GB mutation with a negative stain for IDH1, indicating a poor prognosis with a mean survival of 10-15 months [13]. 

Based on our case and these related articles, there is a suggestion that LZTR1 mutations in schwannomatosis can increase the risk of the development of GB. Surveillance rates in schwannomatosis do not have strict guidelines. Some with symptomatic tumors receive MRI-specific screening yearly. However, asymptomatic tumors are often found in the initial MRI imaging and there is little research on whether regular surveillance is necessary. There have been suggestions that pediatric populations and those with known genetic mutations should be screened every two to three years with asymptomatic tumors [14]. Our case would support the argument for more routine imaging screening in these individuals with LZTR1 mutations.

## Conclusions

Glioblastomas (GB) are associated with neurofibromatosis type 1 (NF1) but limited literature has linked schwannomatosis with glioblastomas, mainly the role of LZTR1 loss of function mutation with the development of GB. Our case may suggest a possible connection between schwannomatosis and GB, and the importance of this connection in caring for patients with schwannomatosis.
